# De novo transcriptome assembly and comparative transcriptomic analysis provide molecular insights into low temperature stress response of *Canarium album*

**DOI:** 10.1038/s41598-021-90011-1

**Published:** 2021-05-18

**Authors:** Ruilian Lai, Xin Feng, Jin Chen, Yongyan Zhang, Xiaoxia Wei, Yiting Chen, Chunzhen Cheng, Rujian Wu

**Affiliations:** 1 Fruit Research Institute, Fujian Academy of Agricultural Sciences, Fuzhou, 350013 China; 2 College of Horticulture, Fujian Agriculture and Forestry University, Fuzhou, 350002 China

**Keywords:** Abiotic, Plant molecular biology

## Abstract

A de novo transcriptome analysis was performed in *C. album,* a temperature sensitive fruit tree in China, after treatment with varied temperatures. A total number of 168,385 transcripts were assembled, comprising of 109,439 unigenes, of which 70,530 were successfully annotated. Compared with control check group (CK), which was treated under 25 °C, the chilling stress (4 °C) treated group (CT), showed about 2810 up-regulated and 2567 down-regulated genes. Whereas, group treated under freezing (− 3 °C) stress (FT) showed an up-regulation and a down-regulation of 1748 and 1459 genes, respectively. GO classification analysis revealed that DEGs related to metabolic processes, single-organism metabolic process, and catalytic activity are significantly enriched in both CT and FT conditions. KEGG pathway enrichment analysis for both CT and FT treatments showed an enrichment of genes encoding or related to glycine/serine and threonine metabolism, alpha-linolenic acid metabolism, carotenoid biosynthesis, photosynthesis-antenna proteins, and circadian rhythm. However, genes related to photosynthesis, carbon fixation in photosynthetic organisms, glutathione metabolism, pyruvate metabolism, nicotinate and nicotinamide metabolism were specifically enriched in CT condition. Nevertheless, FT treatment induced genes related to plant-pathogen interaction, linoleic acid metabolism, plant hormone signal transduction and pentose phosphate pathway. Many of the genes involved in plant hormone signal transduction showed significantly different expression in both FT and CT conditions. However, the change was more evident in FT. Here we present the first of the reports for a de novo transcriptomic analysis in *C. album,* suggesting that the plant shows differential responses in chilling and freezing temperatures, where the hormone signaling and transduction contribute greatly to FT responses. Our study thus paves way for future research regarding functions of these potentially identified genes.

## Introduction

Low temperature (LT) limits the geographical distribution and cultivation of many crops because of plant growth inhibition, substantial reduction in production, and even plant death^1^. Generally, it is divided into chilling injury (0–15 °C) and freezing injury (< 0 °C). Plants originating from temperate regions undergo adaptive resistive acclimation at low, non-freezing temperatures or temperature slightly below freezing point, but severe damages can still be caused under extremely critical LT. However, plants originating from tropical and subtropical regions are usually LT-sensitive. Under LT stress the cell structure, membrane composition, antioxidant component and gene transcription of plant changes significantly causing physiological disorders related to water status, mineral nutrition, photosynthesis, respiratory rate, and metabolism etc.^2^. Cold tolerance in plants is usually controlled by two categories of genes: the functional or structural genes^3,4^ encoding functional proteins that directly respond to LT stress and the regulatory genes^5^ that code for protein kinases and transcription factors regulating the expression or signal transduction of downstream targets of LT responsive genes.

RNA sequencing technology (RNA-Seq) is widely helpful in discovering the molecular mechanisms involved in plant stress responses including changes to metabolism and the relationship between host energy metabolism, signal transduction and various defense responses^6^. It utilizes high throughput analysis that covers a wide range of RNAs and provides high precision too. For many minor crops, the information related to unigenes or expressed sequence tags (ESTs) is very limited, which greatly hinders the research advancement in their case. RNA-Seq, however, being reference genome independent, counts for an economic, effective, and widely used research method for obtaining transcriptomic data for any organism. Recently, with the application of RNA-Seq, research involving transcriptome analysis had been carried out on many plants and great progress has been made in interpreting the LT response mechanisms, screening of key regulatory genes and construction of metabolism regulation network. Many putative genes contributing to the cold stress response in *Rumex patientia*, including the members of *MYB*, *AP2*/*ERF*, *CBF*, *Znf*, *bZIP*, *NAC* and *COR* gene families have been identified^7^. Transcriptional regulation, molecular transport and signal transduction pathways, involved in the adaptation to low temperatures in *Saussurea involucrate* have also been studied using de novo RNA-Seq^8^. Transcription factors like *ARR*-*B*, *bHLH*, *ERF*, *MYB* and *WRKY* etc. are believed to be specifically involved in cold-response in *Hevea brasiliensis*^5^. Moreover, RNA-Seq is successfully been used in discovering LT stress responses in crops like *Oryza sativa, Taxus chinensis,* and *Hordeum vuglare*^9–11^.

*Canarium album*, an evergreen and high nutritional fruit tree, is mainly distributed and cultivated in Fujian, Guangdong, Zhejiang, Guangxi, Sichuan and Chongqing provinces of China. It is also cultivated in a small numbers in north-central Vietnam, Japan and Malay Peninsula. It has great amounts of tannin, polysaccharide, and petroleum ether^12–14^, that show great antioxidant activities. Gallic acid, produced by *C*. *album*, has been proved to have radical scavenging capabilities^15^ and the ethyl acetate and ellagic acid, found in it, can be used for anti-HIV and anti-hepatitis B virus remedies^16,17^. Moreover, *C. album* is also a source of caffeic acid N-nonyl ester showing good anti-bacterial characteristics^18^.

China has a huge market for freshly available and edible fruits of *C*. *album*. However, the *C. album* tree is quite sensitive to LT. − 3 °C is considered to be the most critical low temperature that *C*. *album* can bear^19^. The main *C. album* production areas in China, such as Shanghang, Minqing, Minhou and Youxi counties in Fujian province and Pingyang and Rui’an counties in Zhejiang province, suffer damages from large-scale low-temperature freezing injury in *C*. *album* trees that also cause even the next year's output to plummet^16^. Therefore, LT stress has become one of the most important limiting factors restricting the stable development and expansion of *C*. *album* fruit industry. Hence, development of LT-resistant cultivation technology and selection of LT-resistant rootstock are of utmost importance. However, most of the recent studies on *C*. *album* were focused on the medicinal and chemical constituent excavation and pharmacological activity identification, very few reports concentrated on LT stress responses per say. Most unfortunately, no reference genome and transcriptome data has been published yet. To discover the LT response mechanisms of *C. album*, we first assembled the de novo transcriptome and then compared the transcriptomic differences under − 3 °C (freezing stress treatment, FT), 4 °C (chilling stress treatment, CT) and 25 °C (control check, CK) treatment conditions. Results from our study provide basis for the gene function analysis and are helpful for uncovering the LT response mechanisms in *C. album*.

## Results

### RNA-Seq and transcriptome assembly

As shown in Table 1, the Q_20_ and Q_30_ value of the three cDNA libraries were > 96% and 90%, respectively. The error rate for bases was below 0.02% and the G/C contents were around 43%. A total of 44,734,290, 44,448,624 and 44,987,308 original raw reads were obtained for CK, CT and FT, respectively. After removing low quality reads, 94.40% (6.33 G), 94.14% (6.28 G) and 94.27% (6.36 G) reads were used for further analysis for CK, CT and FT, respectively.

**Table 1:** RNA-Seq results. Q_20_/Q_30_ mean the rate of bases whose *p*-values were more than 20/30.

Sample	Raw reads number	Clean reads number (%)	Clean bases	Error rate (%)	Q_20_(%)	Q_30_(%)	GC content (%)
CK	44,734,290	41,844,672 (94.40)	6.33 G	0.02	96.42	90.85	43.11
CT	44,448,624	42,409,426 (94.14)	6.28 G	0.02	96.52	91.00	42.78
FT	44,987,308	42,230,274 (94.27)	6.36 G	0.02	96.46	90.87	43.17

De novo transcriptome assembly of *C*. *album* highlighted 168,385 transcripts, whose N_50_ and N_90_ values were 2036 bp and 369 bp, respectively. The longest, the shortest and the average length of transcripts were 19,220 bp, 201 bp and 1086 bp, respectively. A total of 109,439 unigenes with an average length of 1517 bp were acquired. The N_50_ and N_90_ of these unigenes was 2543 bp and 648 bp, respectively.

### Unigene annotation

**Figure 1:**
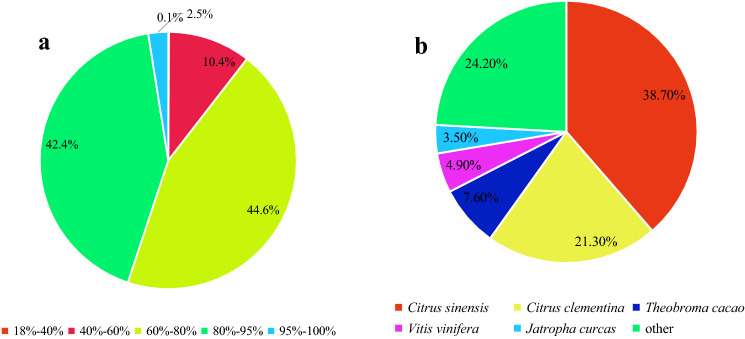
Similarity distribution and species classification of the annotated unigenes. (**a**) The pie-chart shows the similarity distribution of annotated unigenes in the mentioned databases. (**b**) Pie-chart showing the similarity of *C. album* with other species according to annotated unigenes.

The unigenes of *C. album* were annotated by using Nr, Nt, Pfam, KOG, Swiss-Prot, KEGG and GO databases (Table 2). Results showed that 70,530 unigenes, that accounted for almost 64.44% of the total number of unigenes, were annotated in at least one database. The Nr database gave the largest number of unigene annotations (65,274; 59.64%), while those in KEGG database got the least annotations (25,196; 23.02%). In addition, 11,284 (10.31%) unigenes were annotated in all the databases. The largest distribution of annotation similarity ranged from 60 to 80% and 80–95%, accounting for 44.60% and 42.40%, respectively (Fig. 1a). According to RNA-Seq alignment results, *Citrus sinensis*, *C. clementina* and *Theobroma cacao* were the ones having the highest similarity to *C. album* (Fig. 1b).

**Table 2:** Annotation results showing the total number of unigenes and their respective percent share as compared to total number of unigenes.

Item	Number of unigenes	Percentage (%)
Annotated in Nr	65,274	59.64
Annotated in Nt	52,605	48.06
Annotated in KEGG	25,196	23.02
Annotated in Swiss-prot	50,854	46.46
Annotated in Pfam	49,695	45.40
Annotated in GO	50,034	45.71
Annotated in KOG	18,653	17.04
Annotated in all databases	11,284	10.31
Annotated in at least one database	70,530	64.44
Total annotation unigenes	109,439	100

### KOG gene function classification of the *C. album* unigenes

**Figure 2:**
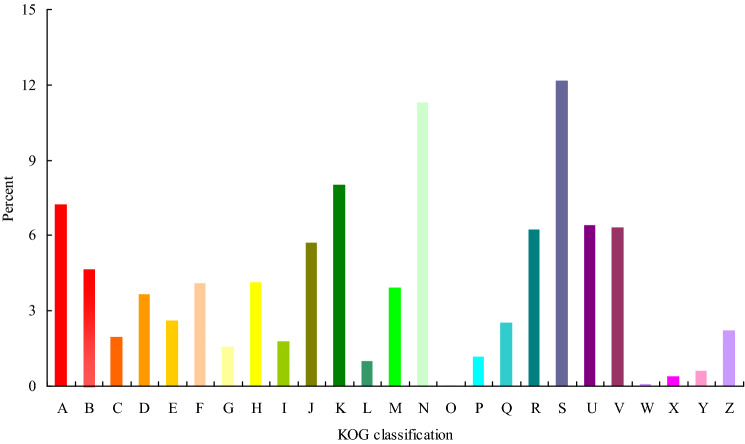
KOG gene function classification of the *C. album* unigenes. **(A)** RNA processing and modification, **(B)** Energy production and conversion, **(C)** Chromatin structure and dynamics, **(D)** Amino acid transport and metabolism, **(E)** Cell cycle control, cell division, chromosome partitioning, **(F)** Carbohydrate transport and metabolism, **(G)** Nucleotide transport and metabolism, **(H)** Lipid transport and metabolism, **(I)** Coenzyme transport and metabolism, **(J)** Transcription, **(K)** Translation, ribosomal structure and biogenesis, **(L)** Cell wall/membrane/envelope biogenesis, **(M)** Replication, recombination and repair, **(N)** Posttranslational modification, protein turnover, chaperones, **(O)** Cell motility, **(P)** Secondary metabolites biosynthesis, transport and catabolism, **(Q)** Inorganic ion transport and metabolism, **(R)** Function unknown, **(S)** General function prediction only, **(T)** Intracellular trafficking, secretion, and vesicular transport, **(U)** Signal transduction mechanisms, **(V)** Extracellular structures, **(W)** Defense mechanisms, **(Y)** Nuclear structure, **(Z)** Cytoskeleton.

The unigenes annotated by KOG were classified into 25 categories (Fig. 2). The proportion of unigenes in general function prediction was the highest with value up to 12.17% of the total unigenes, followed by posttranslational modification, protein turnover, chaperones category (11.30%), translation, ribosomal structure and biogenesis (8.02%) and RNA processing and modification (7.23%) categories. Number of unigenes related to cell mobility were the least in number (0.06%) along with unigenes involved in defense mechanisms (0.07%).

### Gene ontology (GO) analysis of *C. album* unigenes

**Figure 3:**
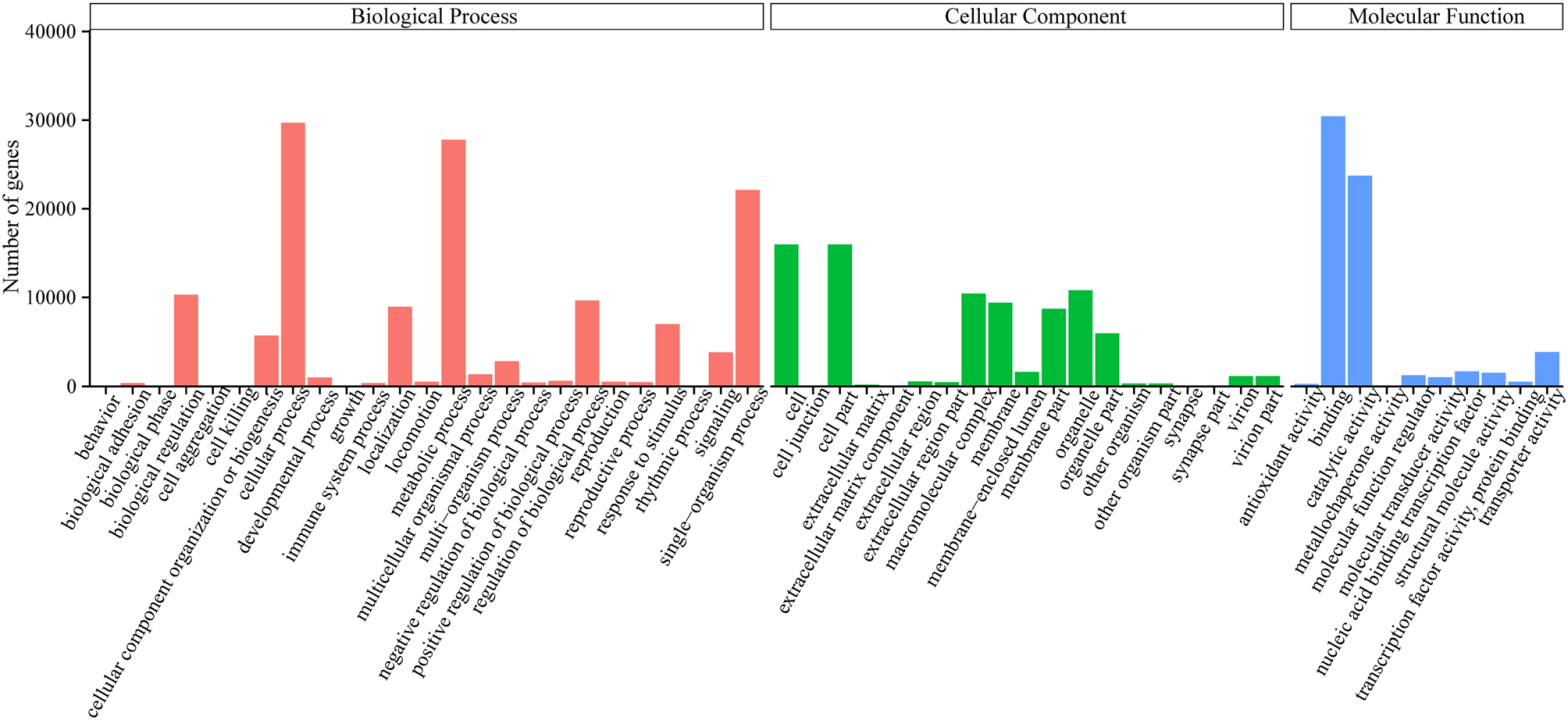
GO gene function classification of *C. album* unigenes. The biological process, the cellular component and the molecular function were shown in red, green and blue, respectively.

GO analysis classified the unigenes from *C. album* in three categories, namely, cellular component (CC), molecular function (MF) and biological process (BP) (Fig. 3). 65.73% of the unigenes were classified into 25 different sub-categories of BP, 19.62% of the unigenes into 19 sub-categories of CC, and 14.65% into 10 sub-categories of MF. Further upon BP analysis we found that majority (top three) of the unigenes were related to cellular, metabolic and single-organism processes, while those for behavior, cell aggregation, growth and rhythmic process were very few in number. In CC, unigenes related to cell and cell parts had the largest share, whereas extracellular matrix component, synapse and synapse part had only a few. The genes related to binding and catalytic activity accounted for the largest proportions in MF and the ones involved in metallochaperone activity comprised a very small proportion.

### KEGG classification of the *C. album* unigenes

**Figure 4:**
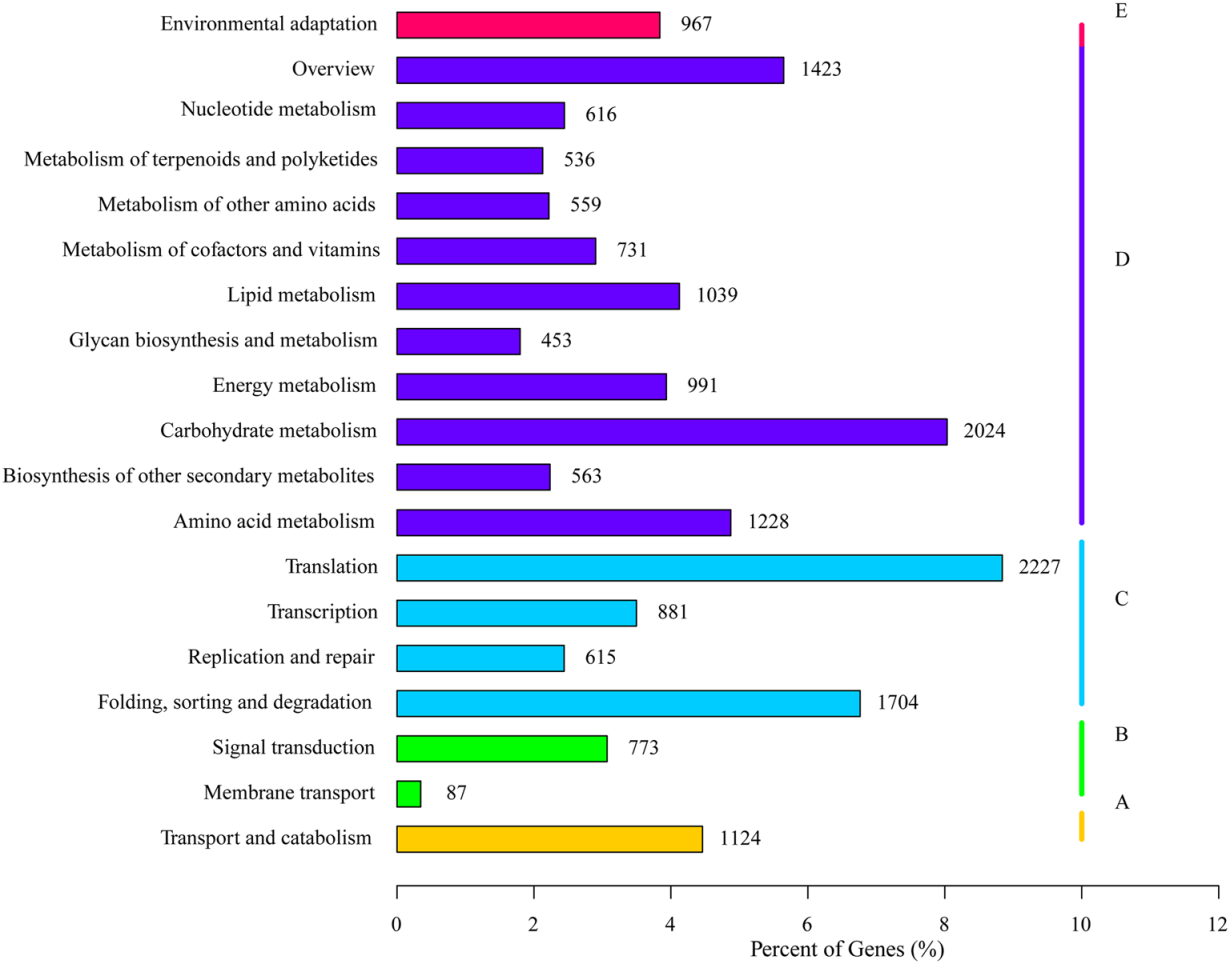
KEGG classifications of unigenes obtained from the *C. album* transcriptomic data. **(A–E)** Indicate the metabolic pathways cellular processes, environmental information processing, genetic information processing, metabolism, and organismal systems, respectively. The numbers represent the unigenes involved in a particular category.

The unigenes identified in *C*. *album* were annotated in 130 KEGG metabolism pathways that belonged to 5 main categories, including those for cellular processes, environmental information processing, genetic information processing, metabolism, and organismal systems (Fig. 4). The greatest number of annotated unigenes were related to metabolism, followed by those involved in genetic information processing. The least number of unigenes belonged to environmental information processing. In the second level of pathway enrichment, the highest number of unigenes were those that are involved in translation and carbohydrate metabolism and the ones involved in membrane transport were the lowest. The top 5 metabolic pathways, based on the number of unigenes involved, were for carbon metabolism, plant-pathogen interaction, ribosome function, amino acid biosynthesis and RNA transport.

### Identification of differentially expressed genes (DEGs) under low temperature treatments

**Figure 5:**
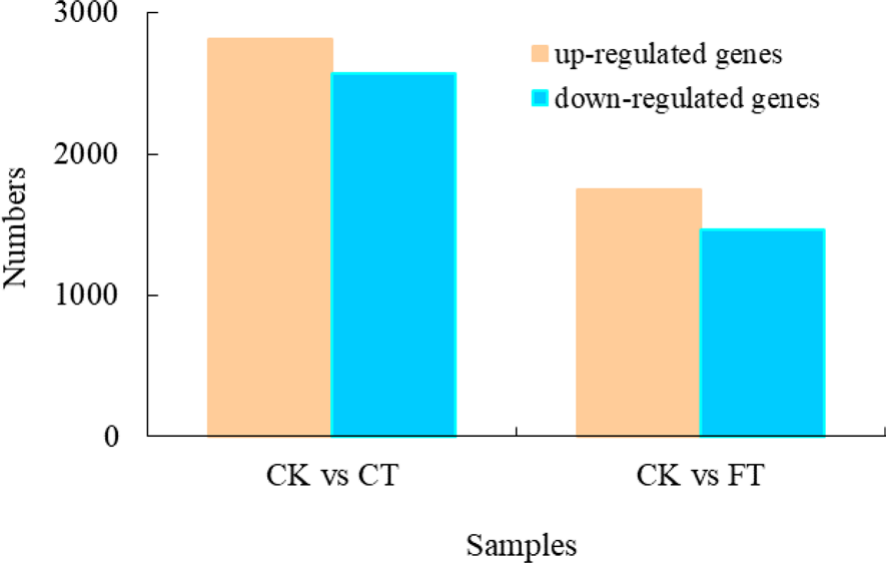
DEGs identified in CT and FT treatments. The graph rep presents the number of genes up and down regulated in CT and FT as compared to CK. The up-regulated genes are shown in orange, and the down-regulated genes in blue.

**Figure 6:**
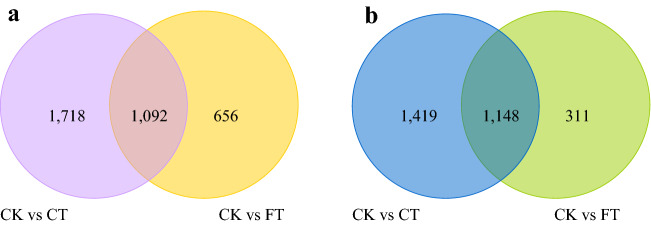
Venn diagram for DEGs. The numbers represent the quantity of DEGs obtained in each category. (**a**) The number of upregulated DEGs in CT and FT compared with CK, and (**b**) The number of down-regulated DEGs in CT and FT compared with CK.

Compared with CK, CT showed 2810 and 2567 genes that were upregulated and downregulated, respectively, while FT resulted in 1748 upregulated and 1459 downregulated ones (Fig. 5). Among these DEGs, 1092 up-regulated and 1148 down-regulated ones were commonly found in both CT and FT (Fig. 6), indicating that they contribute to both chilling and freezing responses in *C*. *album*.

### GO enrichment analysis of DEGs

**Figure 7:**
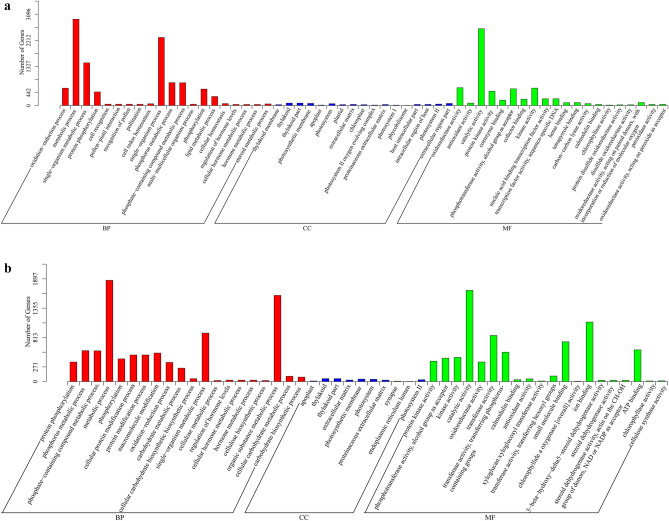
GO classification of DEGs under CT and FT treatments. (**a**) The graph shows the DEGs forming the metabolic pathways that appeared in GO analysis in CT treatment as compared to CK condition. (**b**) Graph showing the DEGs involved in GO classified metabolic pathways in FT treatment as compared to CK treatment. Here CK, CT and FT stand for control check, chilling stress and freezing treatments, respectively.

2250 DEGs identified in CT treatment in BP category of GO, were further annotated and classified into 20 sub-categories, where DEGs involved in metabolic processes were the most abundant ones followed by the ones in single-organism process and single-organism metabolic process (Fig. 7a). 2005 DEGs identified in FT treatment were mainly classified into 20 sub-categories, mostly related to metabolic processes in general, and the metabolic processes related to single organism and organic substances. Processes related to protein phosphorylation, general metabolism, metabolism of phosphorus and phosphate-containing compounds, oxidation–reduction reactions, single-organism metabolic processes, hormonal control, and metabolism, were all found to be enriched in CT and FT treatments (Fig. 7b). Genes involved in cell recognition, pollen-pistil interaction, recognition of pollen, pollination, cell redox homeostasis, single and multicellular organism processes, lipid metabolism, cellular homeostasis and steroid metabolism were specifically enriched in CT. Cellular proteins and other macromolecule modification processes, carbohydrate metabolism and biosynthesis, cellulose metabolism and biosynthesis, metabolism of organic substances, cellular carbohydrate metabolism and biosynthesis were enriched only in FT.

499 and 456 DEGs were enriched in 17 and 10 sub-categories after respective CT and FT treatments. Apoplast, thylakoid, thylakoid part, extracellular matrix, photosynthetic membrane, photosystem, proteinaceous extracellular matrix, photosystem II all were found to be enriched in both CT and FT treatments. The metabolism pathways involving plastid, chloroplast, photosystem II oxygen evolving complex, photosystem I, phycobilisome, host intracellular part, intracellular region of host and extracellular region were found to be strictly CT enriched, while synapse and endoplasmic reticulum lumen were only enriched in FT treatment.

A total of 1108 DEGs after CT treatment fell into 20 sub-categories in MF with the most abundant pathway related to catalytic metabolism. 965 of the observed genes that are involved in catalytic, ion binding, transferase, small molecule binding and ATP binding activities were significantly enriched in FT condition. Protein kinases, phosphotransferases with carboxyl group as acceptor, kinases, catalytic proteins, oxidoreductases, calmodulin binding proteins, antioxidant molecules, and chlorophyllase were all enriched in both, CT and FT conditions. CT specifically enriched the metabolic pathways involving various coenzymes, cofactors, nucleic acid binding transcription factors, transcription factor activity-sequence-specific DNA binding factors, heme binding factors, tetrapyrrole binding factors, carbon–carbon lyases, protein disulfide oxidoreductase, disulfide oxidoreductase and peroxidases. While only FT caused enrichment of various transferases, xyloglucan: xyloglucosyl transferase, small molecule binding factors, chlorophyllide A oxygenase [overall activity], ion binding factors, 3-beta-hydroxy-delta 5-steroid dehydrogenase, steroid dehydrogenase, ATP binding factors and cellulose synthase.

### KEGG pathway analysis of DEGs

**Figure 8:**
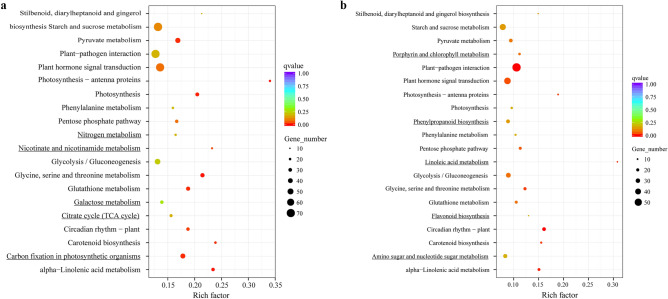
Twenty most enriched pathways in KEGG analysis as based on DEGs. (**a**) Enriched metabolic pathways after KEGG analysis of DEGs in CT treatment as compared to CK. (**b**) Metabolic pathways that showed up after KEGG analysis of DEGs that showed up in FT treatment with comparison to CK. The underlined pathways are specifically enriched in CT or FT when compared with CK.

Compared with CK, the 1912 DEGs in CT treatment were found to be involved in 117 pathways whereas, the 1075 DEGs identified in FT were involved in 109 pathways. Out of the top 20 enriched pathways enriched both in CT and FT 15 pathways (Fig. 8), including photosynthesis-antenna proteins, glycine/serine and threonine metabolism, alpha-linolenic acid metabolism, photosynthesis, glutathione metabolism, carotenoid biosynthesis, pyruvate metabolism, circadian rhythm-plant, plant hormone signal transduction, pentose phosphate pathway, starch and sucrose metabolism, plant-pathogen interaction, stilbenoid/diarylheptanoid and gingerol biosynthesis, phenylalanine metabolism, glycolysis /gluconeogenesis. Besides, five pathways were specifically enriched in CT, including carbon fixation in photosynthetic organisms, nicotinate and nicotinamide metabolism, citrate cycle (TCA cycle), nitrogen metabolism, and galactose metabolism. Pathways related to linoleic acid metabolism, porphyrin, and chlorophyll metabolism, phenylpropanoid biosynthesis, amino and nucleotide sugar metabolism, and flavonoid biosynthesis were found to be enriched in FT only.

Compared with CK, there were 10 metabolic pathways (involving 232 genes) significantly enriched in CT treatment (*p* < 0.005). These metabolic pathways include proteins related to photosynthesis-antenna, glycine/serine, threonine and alpha-linolenic acid metabolism, photosynthesis, carbon fixation in photosynthetic organisms, glutathione metabolism, carotenoid biosynthesis, pyruvate metabolism, circadian rhythm–plant, nicotinate and nicotinamide metabolism (Table 3). In these metabolic pathways, 18 DEGs in photosynthesis-antenna proteins were downregulated. Glycine/serine and threonine metabolism contained 17 down and 18 upregulated genes. Alpha linolenic acid metabolism enrichment showed 11 downregulated and 17 upregulated genes. Among the 32 and 41 DEGs involved in photosynthesis and carbon fixation in photosynthetic organisms, 30 and 29 DEGs, respectively, were inhibited. 22 and 12 genes were down and upregulated respectively, that are involved in glutathione metabolism whereas, 13 and 7 down and upregulated genes are involved in carotenoid biosynthesis. 18 genes were found to be inhibited and 25 gens were induced in Pyruvate metabolism. Among the 28 DEGs of circadian rhythm in plant, 25 DEGs were found to be downregulated. Nicotinate and nicotinamide metabolism contained 7 downregulated genes and 9 upregulated genes. Most of DEGs related to photosynthesis or biological rhythm management were downregulated (accounting for 85.71% of the total DEGs in these metabolic pathways) whereas, other metabolic pathways regulating photosynthesis-antenna proteins, photosynthesis, carbon fixation in photosynthetic organisms, plant circadian rhythm, were found to be inhibited. The photosynthetic capacity of *C*. *album* was found to be decreased after chilling injury, and the biological rhythm adjusted to adapt to the environmental changes. Glutathione metabolism, pyruvate metabolism, nicotinate and nicotinamide metabolism are all closely related to plant antioxidant or oxidative responses. Their significant enrichment after chilling stress indicated that they play important roles in the resistive responses of *C*. *album* towards chilling stress.

**Table 3:** KEGG metabolic pathways significantly enriched in CT treatment as compared with CK.

Metabolism pathways	DEG number	Background number	*p*-value	DEG names
Photosynthesis—antenna proteins	18	53	3.076 × 10^–5^	*LHCA1, LHCA2, LHCA3, LHCA4, LHCA5, LHCB1, LHCB3, LHCB4, LHCB5, LHCB6, LHCB7*
Glycine, serine and threonine metabolism	35	164	5.821 × 10^–5^	*AMT, AOC2, AOC3, betB, DLD, GCSH, GGAT, GLDC, GLYK, gbsA, gcvH, gcvP, gcvT, glyA, gpmB, HPR1, HPR2, ilvA, ltaE, lpd, pdhD, PHGDH, PSAT1, SDS, serA, serC, SHMT, tdcB, thrC, tynA, trpB, SHMT, ltaE, gcvH, GCSH*
alpha-Linolenic acid metabolism	28	120	8.481 × 10^–5^	*ACOX1, ACOX3, ACX, ADH1, AOS, DOX1, HPL, JMT, LOX2S, OPR*
Photosynthesis	32	157	2.437 × 10^–4^	*atpD, atpF, atpG, atpH, ATPF0B, ATPF1B, ATPF1D, ATPF1G, petC, petF, petH, psbC, psaD, psaE, psaF, psaH, psaK, psaL, psaN, psbP, psbQ, psbR, psbS, psbW, psbY, psb27*
Carbon fixation in photosynthetic organisms	41	232	5.082 × 10^–4^	*ALDO, FBP, GAPA, GGAT, glpX-SEBP, GOT1, GOT2, maeB, MDH2, NADP*^+^*, pckA, PGK, ppc, ppdK, PRK, prkB, rbcS, rpe, rpiA, tktA, tktB*
Glutathione metabolism	34	182	6.341 × 10^–4^	*APX, G6PD, GGT1/5, gnd, gntZ, gpX, gshA, gshB, GSS, GST, icd, IDH1, IDH2, ODC1, pepN, PGD, RRM2, spec, speE, speF, SRM, zwf*
Carotenoid biosynthesis	20	84	6.664 × 10^–4^	*ABA1, AOG, CCD8, crtB, crtL1, crtL2, crtP, crtQ, crtY, crtZ, YP707A, CYP97C1, lcyB, lcyE, LUT1, NCED, NPQ1, PDS, VDE, ZDS, ZEP*
Pyruvate metabolism	43	257	1.001 × 10^–3^	*ALDH, accA, aceF, ACSS, atoB, DLAT, DLD, fumB, fumC, GLO1, gloA, ldh, LDHD, lpd, maeB, MDH2, NADP*^+^*, pckA, PDHA, pdhC, pdhD, PK, ppc, ppdK, pyk*
Circadian rhythm—plant	28	150	1.839 × 10^–3^	*APRR1, CHS, CSNK2A, LHY, PHYA, PHYB, PRR5, PRR7, SPA1, TOC1*
Nicotinate and nicotinamide metabolism	16	69	2.775 × 10^–3^	*ENPP1/3, nadA, nadB, NADK, NAPRT1, pncB, ppnK, SDT1, URH1*

Compared with CK, there were 9 significantly enriched metabolic pathways including 219 DEGs in FT treatment. These metabolic pathways include those related to plant-pathogen interaction, plant circadian rhythm, alpha-linolenic acid and linoleic acid metabolism, glycine/serine and threonine metabolism, photosynthesis-antenna proteins, carotenoid biosynthesis, plant hormone signal transduction, and pentose phosphate pathway (Table 4). In these pathways, 52 of the 58 DEGs in plant-pathogen interaction were up-regulated. 20 down-regulated genes and 4 up-regulated genes were involved in plant circadian rhythm. Alpha-linolenic acid metabolism pathway showed 11 down-regulated and 7 up-regulated genes, whereas 7 out of 8 DEGs in linoleic acid metabolism were up-regulated. 10 genes showed up-regulation and 10 were down-regulated in glycine/serine and threonine metabolism. 10 genes involved in photosynthesis-antenna proteins were down- regulated. 6 genes involved in carotenoid biosynthesis were down-regulated and 7 were up-regulated. Plant hormone signal transduction pathway had 23 down-regulated and 22 up-regulated genes. 11 genes were down-regulated whereas, 8 were up-regulated in pentose phosphate pathway.

**Table 4:** Significantly enriched KEGG metabolism pathways in FT treatment as compared with CK.

Metabolism pathways	DEG number	Background number	*p*-value	DEG names
Plant-pathogen interaction	58	560	7.501 × 10^–6^	*BAK1, CALM, CERK1, CML, CNGF, CPK, EDS1, FLS2, GK, glpK, HSP90B, htpG, MEKK1P, PBS1, PR1, PTI1, RBOH, RIN4, RPS2, FLS2, MEKK1P, WRKY2, WRKY33*
Circadian rhythm – plant	24	150	1.024 × 10^–5^	*APRR1, CHS, LHY, PIF3, PRR5, PRR7, SPA1, TOC1*
alpha-Linolenic acid metabolism	18	120	2.583 × 10^–4^	*ACOX1, ACOX3, ACX, ADH1, AOC, AOS, HPL, JMT, LOX2S, OPR*
Linoleic acid metabolism	8	26	2.622 × 10^–4^	*LOX1/5, LOX2S*
Glycine, serine and threonine metabolism	20	164	1.308 × 10^–3^	*AGXT, AMT, AOC2, AOC3, DLD, gcvP, gcvT, GGAT, GLDC, glyA, gpmB, ilvA, lpd, late, pdhD, PHGDH, SDS, sera, SHMT, tdcB, thrA, trpB, tynA*
Photosynthesis—antenna proteins	10	53	1.326 × 10^–3^	*LHCB4 LHCA4 LHCA1 LHCA3 LHCB6 LHCA1 LHCB6 LHCB1 LHCB3 LHCA2*
Carotenoid biosynthesis	13	84	1.383 × 10^–3^	*ABA1, CCD8, crtB, crtZ, NPQ1, VDE, ZEP, CYP707A*
Plant hormone signal transduction	45	524	2.879 × 10^–3^	*ABF, AHK2/3/4, ARF, ARR-B, BAK1, DELLA, EBF1/2, EIN3, ERF1, ERS, ETR, GH3, GID1, IAA, JAR1, NPR1, PIF3, PP2C, PR1, PYL, SAUR, SNRK2, TGA, K14486*
Pentose phosphate pathway	19	169	3.884 × 10^–3^	*ALDO, devB, FBP, G6PD, gapN, gnd, gntZ, PGD, PFK, pfkA, pgl, PGLS, PRPS, prsA, rpiA, zwf*

In these significantly enriched metabolic pathways, those of glycine/serine and threonine metabolism, alpha-linolenic acid metabolism, carotenoid biosynthesis, photosynthesis-antenna proteins, and plant circadian rhythm were significantly enriched in both CT and FT treatments. Photosynthesis, carbon fixation in photosynthetic organisms, glutathione metabolism, pyruvate metabolism, nicotinate and nicotinamide metabolism were specifically enriched in CT condition, while pathways involved in plant-pathogen interaction, linoleic acid metabolism, plant hormone signal transduction, pentose phosphate pathway were specifically enriched in FT treatment. Notably, the DEGs involved in circadian rhythms of plant and photosynthesis-antenna proteins were mostly down-regulated, indicating that both chilling and freezing temperatures inhibited these pathways in *C*. *album*. Although the number of DEGs in alpha linolenic acid metabolism, glycine/serine and threonine metabolism, carotenoid biosynthesis in chilling and freezing stress was large, the overall differences were not significant. However, compared with the chilling stress, the metabolic pathways for glutathione, pyruvate, nicotinate and nicotinamide were not significantly enriched in FT, suggesting that the changes in these metabolic pathways play more important roles in chilling responses of *C*. *album*. Alternatively, freezing treatments caused significant enrichment of genes involved in linoleic acid metabolism, pentose phosphate pathway, and plant hormone signal transduction, indicating that these pathways are involved in resistance to freezing temperature by *C*. *album*. There were especially 45 DEGs serving in pathways related to plant hormone signal transduction, suggesting that the hormones are indeed essential in developing resistance to freezing stress in *C*. *album*.

### Quantitative real time PCR (qRT-PCR) analysis of DEGs

**Figure 9:**
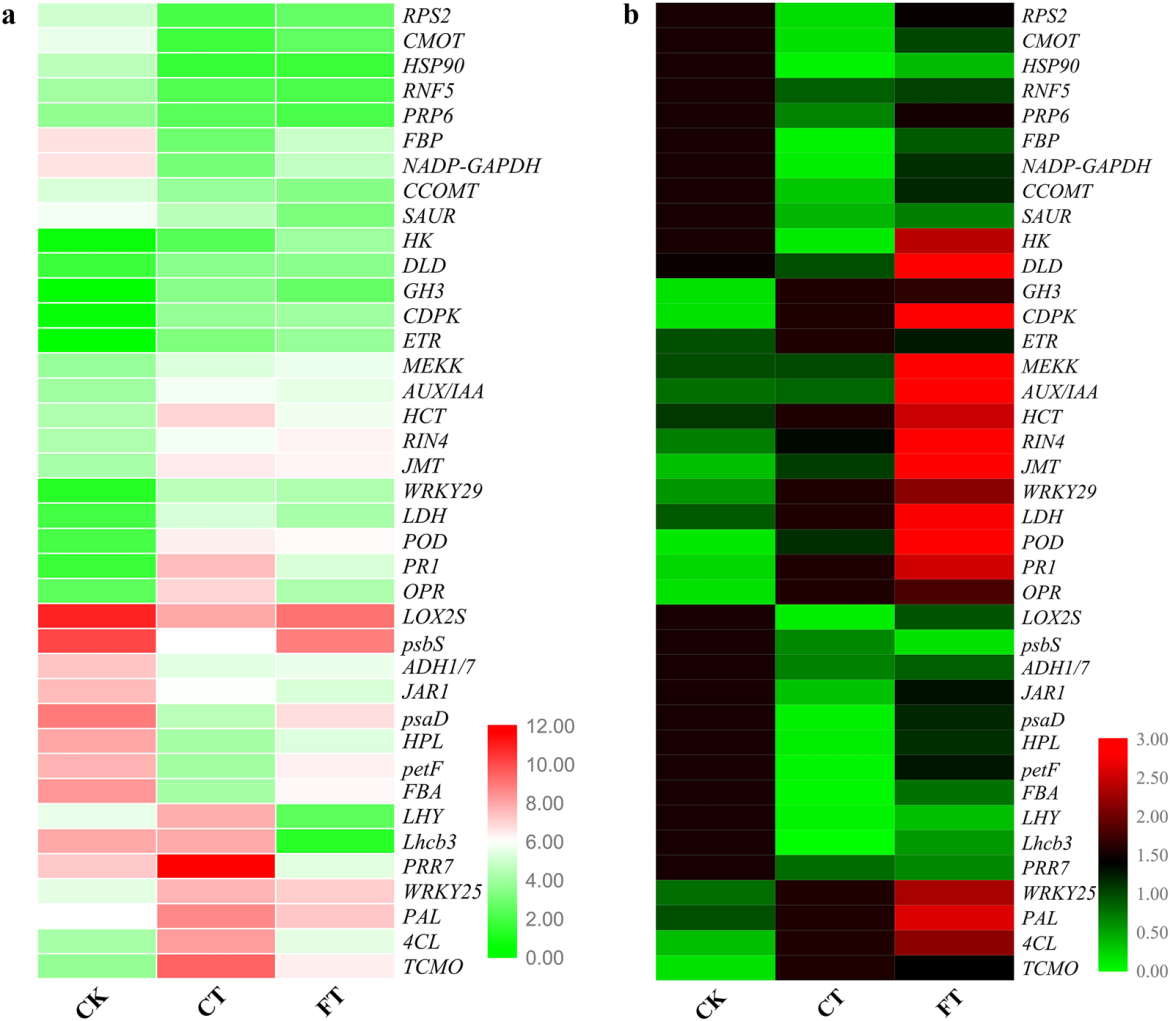
DEGs screening by FPKM value and qRT-PCR analysis. (**a**) The map shows the change in FPKM value of selected DEGs in CK, CT and FT treatments. Each column represents a given treatment and each row indicates relative expression or FPKM value of the gene written on the right side. (**b**) Heat map showing the relative expression of selected DEGs in CK, CT and FT conditions as measured through qRT-PCR. Gene abbreviations are being expanded in Table 5.

The expression levels of 39 genes, selected from enriched metabolic pathways from KEGG analysis were further studied. Compared with CK, the expression levels of 19 genes in CT and FT were significantly increased, while 20 genes were significantly downregulated both in CT and FT treatments (Fig. 9b). The relative expression levels of most of the genes verified by qRT-PCR were consistent with the RNA-Seq data (Fig. 9a) further validating the results obtained in RNA-Seq.

### Verifying the DEGs involved in hormone signal transduction pathways

**Figure 10:**
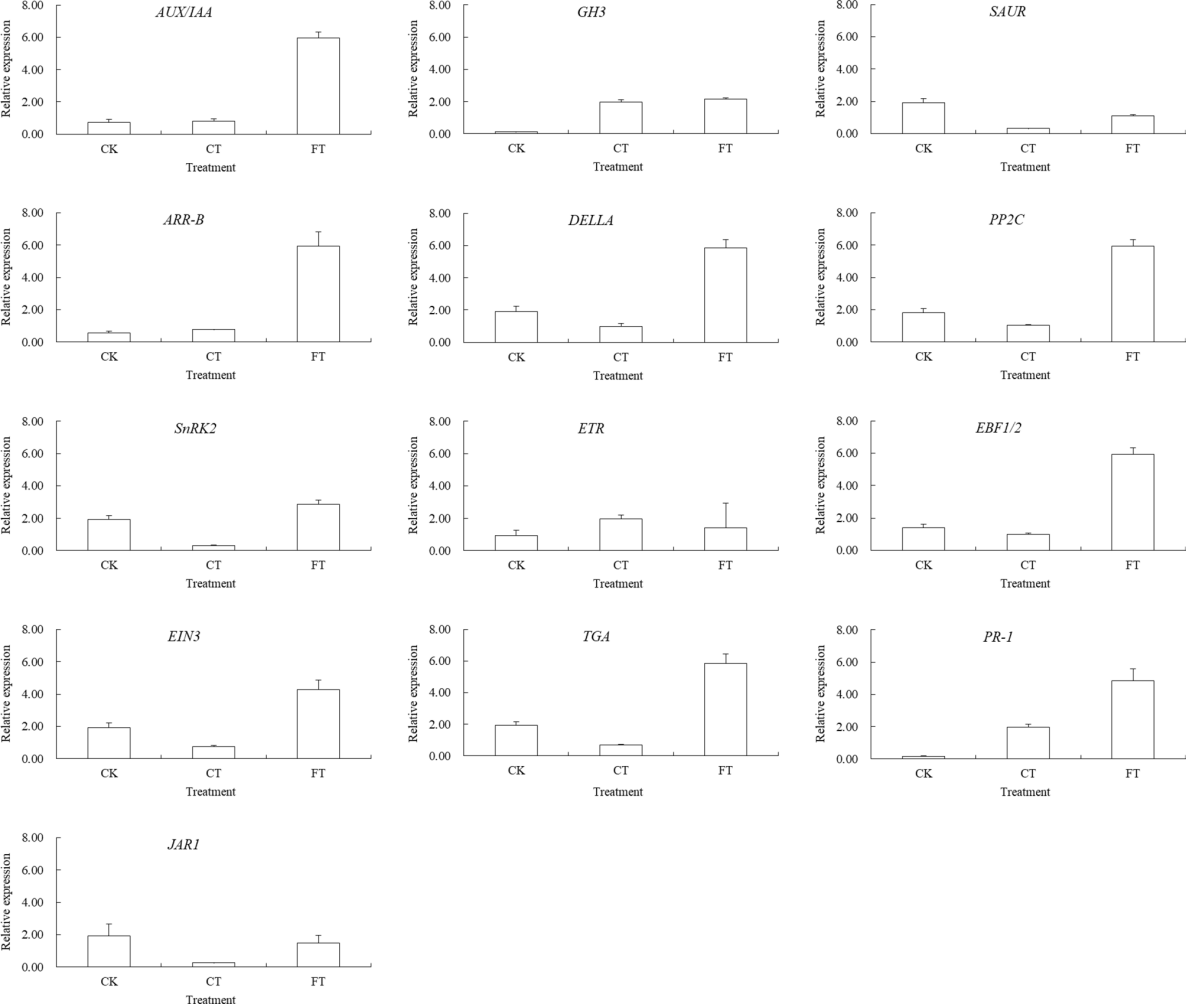
qRT-PCR analysis of some key genes involved plant hormone signal transduction. *AUX*/*IAA* auxin/indole-3-acetic acid, *GH3* Gretchen hagen3, *SAUR* small auxin-up RNA, *ARR*-*B* type-B authentic response regulator, *DELLA* DELLA protein, *PP2C* 2C type protein phosphatases, *SnRK2* sucrose non-fermenting 1-related protein kinases 2, *ETR* ethylene receptor, *EBF1*/*2* EIN3 binding f-boxprotein 1/2, *EIN3* ethylene insensitive 3, *TGA* TGA transcription factors, *PR*-*1* pathogenesis related protein 1, *JAR1* jasmonic acid-amino synthetase 1.

Under low temperature stress, plant hormone signal transduction pathway was significantly enriched in response to freezing injury but not to the chilling injury. The differential expression of 13 key genes from auxin, cytokinin, gibberellin, abscisic acid, ethylene, and salicylic acid signaling pathways were also analyzed by qRT-PCR (Fig. 10). Compared with CK, CT did not significantly change the expression level of *Aux*/*IAA* in auxin signal transduction pathway. But its expression was increased more than eightfold in FT. The expression of *GH3* increased 16 and 18 times respectively, in CT and FT, while the relative expression level of *SAUR* was decreased to about 1/6 and 1/2 of that in CK. Although *ARR*-*B* from cytokinin signal transduction pathway increased a little in CT, the difference was not significant. However, its relative expression level in FT was increased more than 10 folds. A key gene of gibberellin signal transduction pathway, *DELLA* was found to be downregulated in CT, but upregulated, nearly 2 folds, in FT condition. *PP2C* and *SnRK2*, two key genes of abscisic acid signal transduction pathway, had the similar response pattern to low temperature stress, both being down-regulated in CT and up-regulated 3 and 1.5 times, respectively, in CT and FT. *ETR*, *EBF1*/*2* and *EIN3*, three key genes of ethylene signal transduction pathway, also changed expression after exposure to low temperature. The relative expression of *ETR* in CT increased while that in FT did not change significantly. The expression levels of *EBF1*/*2* and *EIN3* consistently decreased in CT but increased 4 and 2 times, respectively, upon FT exposure. *TGA* and *PR*-*1*, the key genes of salicylic acid signal transduction pathway, responded to low temperature stress differently, in which *TGA* was significantly up-regulated (3 times) in CT, while *PR*-*1* increased 11 and 27 times in CT and FT both, respectively. *JAR1* is the key gene of jasmonate signal transduction pathway and the expression level of *JAR1* also changed during low temperature stress in *C. album*. These results further substantiate our findings that auxin, cytokinin, gibberellin, abscisic acid, ethylene, jasmonate and salicylic acid signal transduction pathways are involved in *C. album* responses to low temperature stress, especially to freezing injury.

## Discussion

### De novo assembly of the *C. album* transcriptome

High-throughput transcriptome sequencing, a modern biology research tool, is a common and popular method for screening DEGs, constructing metabolism networks and studying molecular regulation in plants^20,21^. In our study, we firstly reported the transcriptomic analysis of *C*. *album*, where a total of 18.97 G clean data was obtained and the base error rate, Q20, Q30 and, GC content all are in accordance with the established requirements. Moreover, the N_50_ and N_90_ values for assembled and spliced genes were 2,543 bp and 648 bp, respectively. 64.44% of unigenes were annotated in at least one of the databases- Nr, Nt, Pfam, KOG, Swiss-prot, KEGG or GO. Therefore, with this clean transcriptomic data in *C*. *album* we have tried to provide accurate references for future research in this area.

### Comparative transcriptome analysis reveals the responses to low temperature in *C. album*

Three transcriptome libraries of *C*. *album* were constructed and the transcript changes in *C*. *album* under chilling and freezing treatments were studied. Compared with control (CK), 2810 genes were upregulated and 2567 were downregulated after chilling stress treatment (CT). Whereas 1748 and 1459 genes were up and down-regulated, respectively, after freezing stress treatment (FT). The numbers of up-regulated genes in CT and FT were both larger than that of down-regulated genes, indicating that *C*. *album* might improves cold resistance by positive regulation of these genes. Moreover, commonly up-regulated 1092 genes and 1148 down-regulated genes in both in LT and FT treatments were observed. There was a specific up-regulation and down-regulation of 1718 and 1419 genes in CT, whereas in FT the numbers were 656 and 311, respectively, indicating a mutually exclusive response in these cases. In addition, the number exclusive and non-exclusive DEGs in FT were significantly less than that in CT, which could be the reason behind the intolerance of *C*. *album* to freezing injury because of compromised life activities under freezing stress.

### Pathways involved in both chilling and freezing responses in *C. album*

GO classification and KEGG enrichment analysis are widely used tools for transcriptomic research. The chilling and freezing responses of *C. album*, as seen in these analyses separately, showed some similarities but bore differences as well. The GO results showed that the shared responses mostly include DEGs related to various metabolic processes (BP category) including single-organism metabolic process, as well as different molecules catalyzing these processes. In contrast, DEGs solely related to FT, were specifically enriched in organic substance metabolic process (BP category), ion binding, transferase activity, small molecule binding and ATP binding, etc., that particularly regulate freezing acclimation in *C*. *album*. The GO classification results indicated that the responses of *C*. *album* to chilling injury and freezing injury are quite different.

Pathways that were significantly enriched after CT and FT treatments, as seen in KEGG pathway analysis were related to or involved in lysine/serine and threonine metabolism, alpha-linolenic acid metabolism, carotenoid biosynthesis, photosynthesis-antenna proteins, and plant circadian rhythm. However, those specifically enriched after CT are photosynthesis, carbon fixation in photosynthetic organisms, glutathione metabolism, pyruvate metabolism, nicotinate, and nicotinamide metabolism. FT treatment particularly enriched pathways regulating plant-pathogen interaction, linoleic acid metabolism, plant hormone signal transduction and pentose phosphate pathway. These results indicated that some of metabolic pathways were enriched in both the stresses (CT and FT) whereas some were quite exclusive to only one kind of stress exposure.

Previous reports have shown that the changes in pathways controlling plant circadian rhythm could help the plant in adjusting to a change in temperature such as cold stress^22^. This involves increasing expression levels of key genes involved in these pathways^8,22,23^. Serine and threonine protein kinases (STPK) like *SpkB*, *SpkD*, *SpkE* and *SPkG,* that function as transcriptional regulators have also been shown to be involved in responses towards low temperature stresses in different organisms^24^. Plant-pathogen interaction is closely related to cold acclimation of grape plant that is put under cold stress (4 °C). Such a treatment increases expression levels of *CNGC*, *CMLs*, *JAZ1*, *Rboh*, *FLS2*, *BAK*, *MEKK1*, *MKKs*, *RPM1*, *RPS5*, *RIN4*, *PBS1*, *WRKYs*, *PR1* and *MIN7* as a response to cold resistance^25^. The accumulation of linolenic acid in peach fruits improves its tolerance to low temperature stress^26^. Low temperature leads to decrease in CO_2_ absorption in many cold-sensitive plants, resulting in the degradation of photochemical efficiency during photosynthesis^27,28^. Presence of carotenoids affects the cold resistance of plants and may also be related to the changes in auxin content, oxidative damage, and membrane permeability^29^. Plant hormone signal transduction pathways, involving ABA, SA, JA, ETH, GA, IAA and CTK are widely recognized to play important part in cold and disease resistance, heat tolerance and other biological or abiotic stress processes in plants^30^. In addition, linoleic acid, pentose phosphate, glutathione, pyruvate, and nicotinamide^31–35^ have all been shown to be closely related to low temperature stress responses or cold resistance in plants. All these past studies suggest that enrichment of such metabolic or signal transduction pathways are closely related to low temperature stress responses in *C*. *album*.

### The plant hormone metabolism and signal transduction mediate FT tolerance in *C. album*

Plant hormones not only regulate the normal growth and development but are also involved in the stress responses. Many studies performed in plants like *Pyrus ussuriensis, Passiflora edulis*, *Cymbidium goeringii*, *Verbena bonariensis* and *Fragaria ananassa*^36–40^ have shown that plants responses to low temperature are in large mediated by plant hormones.

*Aux*/*IAA*, *GH3* and *SAUR* are the key genes for Aux/IAA-TIR1 nuclear signaling pathway. *Aux*/*IAA* dimers with *Auxin Response Factor* thus inhibiting its transcriptional regulation functions. *GH3* encodes enzymes catalyzing auxin and amino acid coupling, and hence maintain auxin homeostasis through feedback regulation and *SAUR* affects functioning in some highly unstable transcriptional lines. The cold acclimation process in *Arabidopsis thaliana* and low temperature stress in soybean involves changes in the expression levels of auxin signal transduction related gene *Aux*/*IAA*^42,43^. In addition, overexpression of *GH3* in rice increases its cold resistance^44^ and many *PtSAUR* genes in *Populus tomentosa* are downregulated after low temperature stress^45^. Moreover, in rice plant the different members of these enzymatic families mediate different responses to low temperature stress^41^. For example, under low temperatures *IAA9*, *IAA20*, *GH3*-*1*, *GH3*-*8,* and *SAUR21* are upregulated whereas, *IAA10*, *IAA25, and SAUR57* are downregulated. The expression patterns of *IAA9*, *IAA20*, *GH3*-*1*, *GH3*-*8* and *SAUR57* in rice are identical to those induced in *C*. *album* under low temperature stress. Our results are consistent with these old findings further supporting the claim that *Aux*/*IAA*, *GH3* and *SAUR* are indeed involved in low temperature stress responses of *C*. *album*.

Cytokinin is an important plant hormone that regulates plant growth and development as well as responses to abiotic stress though key genes like *ARR*-*A* and *ARR*-*B*. The expression of *ARR18* gene, a positive regulator of low temperature responses, is significantly increased in rubber plant after low temperature treatment^5^. We also found an increased expression level of *ARR-B* gene in *C*. *album* after chilling and freezing stress, which positively correlated with the extent of temperature downregulation.

A protein named DELLA is the core component of gibberellin signal transduction pathway and plays a negative role in regulating GA signal transduction pathway^48^. Gibberellin plays an important role in plant responses to low temperature stress^46,47^. DELLA enables plant survival through adverse environmental conditions by regulating plant hormones^49^. The tolerance of plants to stress depends on the amount of DELLA in vivo. When the active gibberellin is limiting, DELLA level increases and the tolerance to stress improves. In tomato, the crosstalk of PIF4 (*Phytochrome interaction 4*) and DELLA regulates *CBF* transcription and hormone homeostasis during cold stress response by integrating light and temperature signaling and sharing of hormone pathways and transcription regulators thus enabling better adaptation of plants to cold stress^50^. We also found an increased expression of *DELLA* in qRT-PCR after freezing injury, suggesting its role in negatively regulating gibberellin content in vivo in *C. album* to adapt to low temperature stress.

SnRK2 is a plant specific protein kinase, which plays an important role in ABA signal transduction and osmotic stress regulation^51^. Low temperature could induce the *SnRK2* gene in tobacco and wheat^52,53^ and overexpression of *TaSnRK2*.*4* in *Arabidopsis* improves its cold resistance through a series of physiological changes such as reduction in rate of water loss, enhanced stability of cell membrane and improvement in the photosynthetic and osmotic potential^54^. *PP2C* is a serine/threonine residue protein phosphatase and a core negative regulator of ABA signal transduction. In *Arabidopsis*, *PP2C* has also been shown to be closely related to the low temperature acclimation^55^. An enrichment of ABA signaling pathway in our study further confirms its involvement in low temperature stress responses and subsequently validates our results too.

Being the key enzyme for jasmonate signal transduction pathway, JAR1 catalyzes the last step forming molecularly active JA-Ile from the combination of jasmonate and amino acids, especially isoleucine^56^. LOX, AOS and JMT are other key enzymes in jasmonate biosynthesis or signal transduction pathway. Previous studies have shown that low temperature could induce the expression of *LOX*, *AOC* and *JAR1* genes in *Artemisia annua*, thus increasing the level of endogenous jasmonate, which consequently induces the expression of *ETR1*, *ETR2* and *AP2*/*ERF*^57^. We also found an increased expression of these genes in *C*. *album*, as response to chilling or freezing injury.

*ETR*, *EBF1*/*2* and *EIN3* are genes serving in ethylene signal transduction pathway. After cold stress in kiwifruit and papaya, expression levels of some of the *ETR* and *EIN3* members are increased, with a slight change in the expression level of *EBF* as well^58,59^. As a key member of salicylic acid signal transduction pathway, transcription factor *TGA* is involved in temperature stress responses in some plants^60^, while *PR*-*1* gets expressed in others^61^. Our results corroborate with these earlier findings further strengthening the role of ethylene and salicylic acid signal transduction pathways in cold stress responses in *C*. *album*.

## Materials and methods

### Plant materials and low temperature treatments

**Figure 11:**
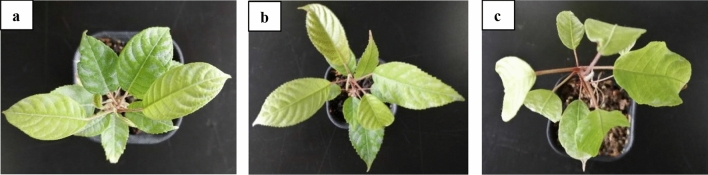
Seedlings of *C. album* treated at different temperatures for 24 h. (**a–c**)show the seedlings of *C. album* that were given treatments for control check (25 °C), chilling stress, CT (4 °C) and freezing stress, FT (− 3 °C) for 24 h, respectively.

Healthy, strong and uniform *C. album*. cv. ‘Fulan 1’ seedlings of height of ~ 20 cm were selected and kept in growth chambers (GXZ-280C, Ningbo, China) maintained at 25 °C, 60% to 80% relative humidity, and a photoperiod of 12 h (2000 ± 200 lx) for one week. Then the seedlings were subjected to three different temperature treatments, i.e. freezing stress treatment (FT, − 3 °C) in freezer, chilling stress treatment (CT, 4 °C) in freezer and control check treatment (CK, 25 °C) in growth chambers for 24 h. Ten seedlings were collected for each treatment and each experiment was repeated 3 times. During the whole treatment process, all seedlings were grown in the same conditions as formal, and the samples were simultaneously collected in dark after treatment. Compared with the seedlings in CK (Fig. 11a), the leaves from seedlings in FT experienced dehydration and gradually wilted (Fig. 11c), while there was no obvious phenotypic change in leaves from seedlings grown in CT (Fig. 11b). After the 24 h temperature treatment of the seedlings, the 3rd and 4th leaves from top to base were collected (from all the replicative experiments) and instantly frozen in liquid nitrogen. The samples were stored in the freezer at -80 °C till further use. The collection of plant material in this research, complied with relevant institutional, national, and international guidelines and legislation.

### Total RNA extraction and detection

Total RNA was extracted by E.Z.N.A. Plant RNA Kit (OMEGA bio-tek, USA) as per the manufacturer’s instructions. The RNA was checked for quality and quantity by first running it on a 1.5% agarose gel, followed by a test for purity, concentration and integrity using the TU-1810 spectrophotometer (Puxi, CHN), NanoDrop OneC Flurometer (Thermo Fisher Scientific, USA) and the Agilent Bioanalyzer 2100 system (Agilent Technologies, USA). Only high-quality total RNA was used for further experiments including the library construction.

### Library preparation for RNA-Seq

1.5 µg of high-quality total RNA, treated at different temperatures was used for library preparation and NEBNext Ultra RNA Library Prep Kit for Illumina (NEB, USA) was used to generate the sequencing libraries and the library preparation and RNA-Seq were carried out by Novogene, Beijing, China^62^. Briefly, mRNA was made from the total RNA using poly-T oligo-attached magnetic beads followed by first strand cDNA synthesis using random hexamer primer and M-MuLV Reverse Transcriptase (RNase H-) as per the manufacturer’s instructions. DNA polymerase I and RNase H were subsequently added for the synthesis of second strand cDNA and the removal of mRNA template. Remaining overhangs were converted into blunt ends by exonuclease/polymerase reactions. After adenylation of 3’ ends of the generated cDNA fragments, NEBNext Adaptor, with hairpin loop structure, were prepared for hybridization. AMPure XP system (Beckman Coulter, USA) was used to purify library fragments where all 150–200 bp cDNA fragments were selected. Then, 3 µL of size selected USER Enzyme (NEB, USA) was used for adaptor-ligation of cDNA at 37 °C for 15 min followed by 5 min of heating at 95 °C before PCR analysis. PCR was performed with Phusion High-Fidelity DNA polymerase, Universal PCR primers and Index (X) Primer according to the mentioned instructions from the manufacturer. Lastly, PCR products were purified by AMPure XP system and library quality was evaluated on Agilent Bioanalyzer 2100 system.

### Clustering, sequencing, and data analysis

Clustering of the indexed-coded samples was done via cBot Cluster Generation System that uses TruSeq PE Cluster Kit v3-cBot-HS from Illumina. The library preparations were sequenced on Illumina Hiseq-2000 platform and paired-end reads were generated after the cluster generation. Followed processing of fastq formatted raw data (raw reads) by internal perl scripts, and clean data (clean reads) were obtained by removing reads with adapter, ploy-N and low-quality raw data. Subsequently, Q_20_, Q_30_, GC-content and sequence duplication level for the clean data were calculated. The high-quality clean data were then used for downstream analysis. Transcriptome was assembled using Trinity with min_kmer_cov default value of 2 and all other parameters were set to their respective default values^63^.

Gene function was annotated using Nr, Nt, Pfam, KOG, Swiss-Prot, KEGG and GO databases. The read counts for each sequenced library was adjusted by edgeR program package through one scaling normalized factor. Differential expression analysis of two samples was performed using the DEGseq R package as shown previously^64^. P value was adjusted by using the q value^65^. *q*-value < 0.005 and |log_2_^(fold change)^|> 1 was set as the threshold for calculating significantly differential expression of a gene.

GO enrichment analysis of the differentially expressed genes (DEGs) was performed according to the GOseq R packages based Wallenius non-central hyper-geometric distribution^22^. The statistical enrichment of DEGs in KEGG pathways was tested by KOBAS software as shown previously^66,67^.

### qRT-PCR analysis of DEGs

TransScript All-in-One First-Strand cDNA Synthesis SuperMix kit (TransGen Biotech, CHN) was used to synthsize high quality cDNA for qRT-PCR analysis. cDNA samples of CK, CT and FT were mixed equally, and then gradiently diluted 4 times to obtain a standard curve (dilutions: 10^–1^, 40^–1^, 160^–1^ and 640^–1^) using a suitable annealing temperature, that was screened according to the amplification efficiency. Eppendorf Realplex 4 and TransStart Top Green qPCR SuperMix were used to detect the relative expression of the selected 39 genes (including 13 plant hormone signaling and transduction pathway related genes, i.e. *AUX/IAA*, *GH3* and *SAUR* involving in auxin signaling pathway, *ARR*-*B* involving in cytokinin signaling pathway, *DELLA* involving in gibberellin signaling pathway, *PP2C* and *SnRK2* involving in abscisic acid signaling pathway, *ETR*, *EBF1*/*2* and *EIN3* involving in ethylene signaling pathway, *TGA* and *PR*-*1* involving in salicylic acid signaling pathway and *JAR1* involving in jasmonate signaling pathway) in different treatments by using primers as listed in Table 5. The amplification cycle of qRT-PCR: pre-denaturation at 94 °C for 30 s; denaturation at 94 °C for 10 s, annealing at 58–60 °C for 15 s, extension at 72 °C for 10 s, 40 cycles. The sample was maintained 94 °C for 15 s, at 60 °C for 15 s, and then the temperature was raised to 94 °C for 15 s at the rate of 0.11 °C/s to draw the dissolution curve. The reaction was set as with 10 μL 2 × TransStart Top Green qPCR SuperMix, 0.4 μL Passive Reference Dye (50 ×), 0.2 μM each upstream and downstream primer, and 0.5 μM cDNA template. *ACTB7* and *TUB5* were used as reference genes^68^. SPSS19.0 was used for analyzing the results statistically. TBtools was used to draw heatmaps according to the relative expression and FPKM value of the genes.

**Table 5:** List of qRT-PCR primers used in the study.

Gene ID	Gene name	Primer sequence (5’–3’)	Description
Cluster-7340.51676	*PRR7*	ATCACAACAGCAGCCTCCT	Pseudo-response regulator 7
		TTCCTGAGCAAAGCACTGAC	
Cluster-7340.66287	*LHY*	AAATCTTCCCACCTCCGAG	Late elongated hypocoty
		CCCTCTTCAGAAACTTCCTTCC	
Cluster-7340.85283	*Lhcb3*	ACCCTCTTAGAAATGTTGCCTC	Light-harvesting chlorophyll a/B-protein
		TGTCCCATCCATAGTCACCTG	
Cluster-7340.42050	*DLD*	TGTTGTTCTTGTCTCCGCTG	Dihydrolipoamide dehydrogenase
		CCATCCTCTTCTGCTTTGTG	
Cluster-7340.48264	*CDPK*	CAACGGAGACTTGACATTTGAC	Calcium-dependent protein kinase
		GCTTCCAGTAACATTCGCAC	
Cluster-7340.41917	*MEKK*	GGAATGTATGCCAGCGTTG	Mitogen-activated protein kinase kinase kinase
		CCTCTTCACAAATGGATGGTC	
Cluster-7340.50488	*WRKY25*	CCATCTCTCCTCCTCCTGTT	WRKY transcription factor 25
		GGTTATCCCAGTTGAAGGTTTG	
Cluster-7340.38428	*WRKY29*	CAGATGTAGCAGTTCAAAGGGT	WRKY transcription factor 29
		AGTGAGTTTCGGTGGGTAGG	
Cluster-7340.58469	*RIN4*	ATGGGAAGGGAAGGGTTT	RPM1-interacting protein 4
		CATCAGGACTTTCATCGCC	
Cluster-7340.37043	*HSP90*	CGATGATGAAGATGATGAGCC	Heat shock protein 90
		CCTCCTTGGTGATTTCCTCTG	
Cluster-7340.74583	*RPS2*	GAAATCTTATGCCAACCCTACC	Resistance to P. syringae 2
		TCCGTCATCACATCCGTTT	
Cluster-7340.51547	*LOX2S*	CAAGTTCAGCCAAGACCCTCT	Lipoxygenase
		CAACAGCCGTAACCTTCAAAG	
Cluster-7340.55174	*HPL*	GAAACTCTCCGACTCAACCC	Hydroperoxide lyase
		ATACCCACAAAGCAACTCTCC	
Cluster-7340.50441	*AOS*	GGTTTGTTGGTGGAGAAGGT	9-allene oxide synthase
		CACTGTTTATTCCCTGCGG	
Cluster-7340.57514	*OPR*	GGCTATCTCCCTTTGCTGAC	12-oxo-phytodienoic acid reductase
		TTTCATTCTGGGCTCAACC	
Cluster-7340.40449	*JMT*	CGGTTCTTTCTATGGCAGG	Jasmonate O-methyltransferase
		TGTGCTGGCGATGTAAATG	
Cluster-7340.58766	*psbS*	TCTCTCAACTTCTGCTCAGTCC	Photosystem I subunit II
		CCATCTTCAACCTTAGGCTTAG	
Cluster-7340.38192	*psaD*	AGTCCCAAACATCTCACCTTC	Photosystem I subunit II
		TCCAGTGCTTCCTCCAAAG	
Cluster-7340.46508	*petF*	GCCGTTACAAGCCTCAAGTC	Protein–protein interaction of GcpE protein with ferredoxin I
		CTCAGCGTGGTCAAGAATGT	
Cluster-7340.50425	*HCT*	TCCCAGCGTCTACTTCTATCG	Shikimate p-hydroxycinnamoyl transferase
		ATCCCTCTTCAACCTTCCG	
Cluster-7340.49158	*CCOMT*	AACCACTACCAGCACTTGATTC	Caffeoyl-CoA O-methyltransferase
		GCAGTAACATCCCTCATCTCC	
Cluster-7340.49770	*POD*	GAGTTGCTGCCTTCATTCTT	Peroxidase
		GCTGCTTCCACCTGAGTTT	
Cluster-7340.3616	*CMOT*	TGTATCGCCAACTGAAATCG	2-(4-chloro-3-methylphenyl)-2-oxoethyl thiocyanate
		GGCTACGCAAAGAGCAAGT	
Cluster-7340.51517	*4CL*	AAACAAGGGTGGCTACATACG	4-coumarate–CoA ligase
		CATCTTTCATTGGGACTACTGC	
Cluster-7340.50851	*TCMO*	ACATCCTCCTTCTCCGCAT	Trans-cinnamate 4-monooxygenase
		CCTCCTCATCTTCCTCCAAT	
Cluster-7340.50096	*PAL*	CCCAAGAAGCCTCCAAACT	Phenylalanine ammonia-lyase
		AACACAGCCAGAACATTAGCC	
Cluster-7340.66106	*HK*	TGCATGAGGATGACACTCC	Hexokinase
		CCAAGGAAGGAGACAAGCA	
Cluster-7340.18603	*FBP*	AAGTTCGTCTGCTCTGCTGTT	Fructose-1,6-bisphosphatase
		GTTGCCTCTTCATCTTCTTCAG	
Cluster-7340.50923	*FBA*	CCCATCTGCCCTTACTGTTC	Fructose-bisphosphate aldolase
		GGTTAGCCTCGGTGTTCTCT	
Cluster-7340.66723	*NADP-GAPDH*	TTTAGCCATCCCACCGTT	NADP-glyceraldehyde-3-phosphate dehydrogenase
		GAGCCTTTCCCAGTGATACAG	
Cluster-7340.43174	*LDH*	AATCCACCCAGTTTCAGTCC	Lactate dehydrogenase
		CGTTTAGCCTCCTCGTCACT	
Cluster-7340.37158	*ADH1/7*	CCTCTACACATCCCTTTGCC	Alcohol dehydrogenase
		AACGCTCTCCACAATCCCT	
Cluster-7340.33488	*AUX/IAA*	AGGAGGACGAGGATGTTACTTT	Auxin/indole-3-acetic acid
		CTGTCAACGGAAGAGCAGTC	
Cluster-7340.64060	*GH3*	GCTGTTGATTCTGCCCTTTC	Gretchen hagen 3
		GCGTTTCGGCTTAGGATTT	
Cluster-7340.51543	*SAUR*	TGCTCTGCTAACTCCAAATCC	Small auxin-up RNA
		ACTCCCTTCTGCTCGTATCC	
Cluster-7340.49359	*ETR*	TTGACCCTATCTTCTCGGTTG	Ethylene receptor
		CAATCCAAATGTGACCTCCC	
Cluster-7340.53982	*JAR1*	AATGTATTCCGCAGTTCCC	Jasmonic acid-amino synthetase 1
		TAAGCCCACAGAGGAGATGG	
Cluster-7340.26872	*RNF5*	ATCCCTCCCGTTGTCTTCT	E3 ubiquitin-protein ligase
		GCGACCTGCTTGTTGATTC	
Cluster-7340.44794	*PRP6*	CCGTTCTGCCACCACTATT	Pre-mRNA-processing factor 6
		CGGCTTCTTTATCATCCTCGT	
Cluster-7340.72327	*ARR-B*	TCAACAAAGTGCTGGCGT	Type-B authentic response regulator
		CATTCTCCTGTCCATCCTCTT	
Cluster-7340.59249	*DELLA*	TGAGCAAGAGACGAACCATAAC	DELLA protein
		GCTTCCCTAAATACACCTCGG	
Cluster-7340.60183	*PP2C*	TTGTTCTCCCTCGGTTCAT	2C type protein phosphatases
		TCCCATTCAAACTCACTCTGAC	
Cluster-7340.20010	*SnRK2*	GGCAACAATAATGGACCGT	Sucrose non-fermenting 1-related protein kinases 2
		TGGTTCATAATCTCCCTCTGC	
Cluster-7340.50447	*TGA*	GGTCAATCTACTGCTATCGTGG	TGA transcription factors
		CTTTCTTCCTCAATCGGCTT	
Cluster-7340.60183	*EBF1/2*	CATAGCCTGTGGATGTCCGT	EIN3-binding F-box protein
		AAGGGCATTGGCAAAGTTC	
Cluster-7340.50747	*EIN3*	GAAGAAGATGTCCCGTGCTC	Ethylene-insensitive protein 3
		ATTATCAGATGCTCCACTCACC	
Cluster-7340.43403	*PR1*	GGCACTACACTCAGGTGGTTT	Pathogenesis-related protein 1
		GCATCCAATGAAAGTTCCTCC	
